# Patient‐oriented medication education intervention has long‐term benefits for people with decompensated cirrhosis

**DOI:** 10.1002/hep4.1999

**Published:** 2022-05-13

**Authors:** Kelly L. Hayward, Vikas Bansal, Patricia C. Valery, Katharine M. Irvine, Penny L. Wright, Caroline J. Tallis, Katherine A. Stuart, W. Neil Cottrell, Jennifer H. Martin, Elizabeth E. Powell

**Affiliations:** ^1^ Centre for Liver Disease Research, Translational Research Institute The University of Queensland Woolloongabba, Brisbane Queensland Australia; ^2^ Department of Gastroenterology and Hepatology Princess Alexandra Hospital Woolloongabba, Brisbane Queensland Australia; ^3^ QIMR Berghofer Medical Research Institute Herston, Brisbane Queensland Australia; ^4^ Mater Research, Translational Research Institute The University of Queensland Woolloongabba, Brisbane Queensland Australia; ^5^ Faculty of Health and Behavioural Sciences The University of Queensland St Lucia, Brisbane Queensland Australia; ^6^ School of Medicine and Public Health University of Newcastle Callaghan, Newcastle Queensland Australia

**Abbreviations**
aIRR: adjusted incidence rate ratio
CI: confidence interval

To the editor,

Patient barriers to self‐management in cirrhosis are complex.^[^ ^1^ ^]^ Although several studies have demonstrated improved knowledge or self‐care behaviors following education,^[^ ^1^ ^]^ long‐term outcome data are lacking. We previously reported that a multifaceted pharmacist‐led intervention for people with decompensated cirrhosis (including patient‐oriented education, medication reconciliation, and identification and resolution of “high‐risk” medication‐related problems) was associated with fewer unplanned admissions at 12 months compared to usual care (adjusted incidence rate ratio [aIRR], 0.52; 95% confidence interval [CI], 0.30–0.92; *p* = 0.025).^[^ ^2^ ^]^ Despite the relatively short 6‐month intervention delivery period, new outcome data demonstrate unplanned admission rates in the intervention group remained lower than usual care out to 3 years.

Participants were followed for a mean of 27.8 (SD, ±11.9) months and censored at death (*n* = 41; 35.3%), liver transplant (*n* = 5; 4.3%), or study closeout at 3 years (*n* = 70; 60.3%). Forty‐one patients receiving intervention (41/57, 71.9%) and 50 patients receiving usual care (50/59, 84.7%) had at least one unplanned admission during the follow‐up period (unplanned admission rate, 3.72 vs. 4.34; Mann‐Whitney U, *p* = 0.272) (Figure 1). Using a generalized linear model with negative binomial distribution offset by person time to censorship, patients receiving intervention had a significantly lower incidence rate of unplanned admissions at 3 years compared to usual care (aIRR, 0.56; 95% CI, 0.34–0.92; *p* = 0.023), following adjustment for Child‐Pugh score (aIRR, 1.47; 95% CI, 1.28–1.68; *p* < 0.001), number of medications (aIRR, 1.09; 95% CI, 1.03–1.15; *p* = 0.005), history of variceal bleeding (aIRR, 2.36; 95% CI, 1.30–4.29; *p* = 0.005), and alcoholic liver disease (aIRR, 0.63; 95% CI, 0.40–1.00; *p* = 0.050).

**FIGURE 1:**
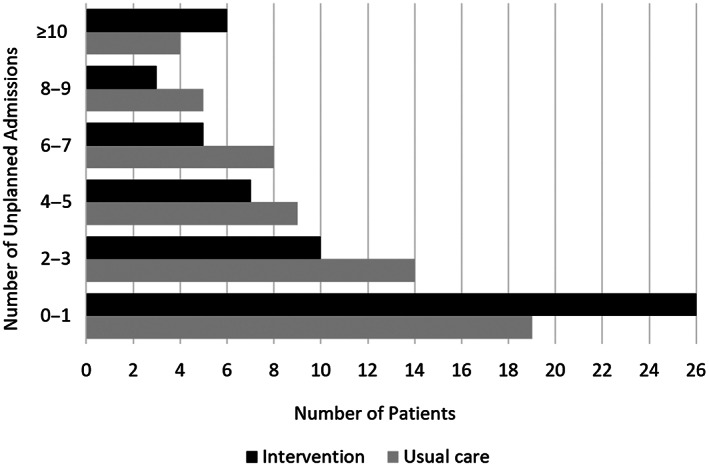
Number of unplanned admissions over 3 years among participants in a randomized‐controlled trial of a pharmacist‐led education intervention for patients with decompensated cirrhosis

We further conducted a subanalysis of unplanned admissions that were considered medication related and potentially preventable.^[^ ^2^ ^]^ Using a multinomial logistic regression model (due to the large count of zero admissions) adjusted for Child‐Pugh score and number of medications, patients receiving usual care were 3.5 times more likely to have three or more “potentially preventable medication‐related admissions” compared to zero than patients receiving intervention, although this did not reach statistical significance (adjusted odds ratio, 3.52; 95% CI, 0.69–17.81; *p* = 0.129). There was no difference in mortality rate (intervention 31.6% vs. usual care 39.0%; *p* = 0.404) or mean time to censorship (28.5 vs. 27.2 months, respectively; log‐rank *p* = 0.530).

These findings suggest that a pharmacist‐led intervention, which empowers patients (and their caregivers) with improved knowledge about cirrhosis and medicines,^[^ ^3^ ^]^ reinforces perceptions of treatment necessity and utility in disease management,^[^ ^3^ ^]^ provides tools to manage medicines (e.g., a structured list^[^ ^2^ ^]^), and builds confidence to actively engage with health care providers, has long‐term benefits for patients and the health care system. Despite completion of active pharmacist intervention at 6 months, the benefits persisted over time and translated to fewer hospitalizations independently of liver disease severity. These data support inclusion of a pharmacist in the multidisciplinary team.

## CONFLICT OF INTEREST

Nothing to report.
